# Immediate one-stage subcutaneous breast reconstruction without ADM: A single-center 6-year experience

**DOI:** 10.1016/j.jpra.2026.02.009

**Published:** 2026-02-08

**Authors:** Antonio Russo, Antonio Taveggia, Francesco Buttarelli, Benedetta Agnelli, Ginevra Sofia Puglisi, Igor Valtorta, Liah Moalem, Luigi Antonio Valdatta, Federico Tamborini

**Affiliations:** a Ospedale Maggiore della Carità di Novara, Plastic and Reconstructive Surgery Departement, Via Gibellini 28100, Novara, Italy; b University of Insubria, Plastic and Reconstructive Surgery Departement, Via Ravasi 2, 21100, Varese, Italy

**Keywords:** Acellular dermal matrix, Capsular contracture, Prepectoral breast reconstruction, Radiation therapy, Surgical outcomes

## Abstract

Prepectoral breast reconstruction without acellular dermal matrix (ADM) is gaining attention as a potentially safe and effective approach, particularly in non-irradiated patients. This study aimed to evaluate the safety and efficacy of immediate one-stage prepectoral breast reconstruction without ADM and assess the impact of radiation therapy (RT) on postoperative outcomes. A retrospective review of 198 patients (239 breasts) who underwent mastectomy followed by direct-to-implant prepectoral reconstruction without ADM between 2018 and 2023 was performed. Patients were divided into RT and non-RT groups. Demographics, complications, and secondary procedures were analyzed. Fisher’s exact tests were used for statistical correlation. In the non-RT group, complication rates were low, with capsular contracture observed in 6 patients (3.5%). In the RT group, capsular contracture was significantly higher (*p* < 0.0001). Other complications were comparable between groups. Secondary procedures were more frequent in the RT group. Immediate prepectoral breast reconstruction without ADM is a safe and effective option, especially in non-irradiated patients, with acceptable complication rates and favorable surgical outcomes.

## Introduction

Breast reconstruction has become an integral part of post-mastectomy care, offering substantial psychological and aesthetic benefits to patients undergoing treatment for breast cancer. Surgical approaches in this field have undergone continuous evolution, with the aim of improving both functional and cosmetic outcomes.

Among these techniques, immediate one-stage prepectoral implant-based reconstruction has recently seen a resurgence in popularity. This renewed interest is largely attributed to the introduction and widespread use of acellular dermal matrices (ADMs), which have expanded reconstructive possibilities and facilitated more reliable implant placement in the subcutaneous plane.

Historically, the concept of subcutaneous or prepectoral implant positioning was introduced in the 1970s,^1^ but its initial implementation was hindered by high rates of complications, including flap necrosis, implant extrusion, and capsular contracture. As a result, the focus shifted for decades toward retropectoral techniques and staged reconstructions with tissue expanders, which were perceived as safer alternatives.

In recent years, however, improvements in surgical technique, implant design, and intraoperative tools for flap evaluation have revived interest in prepectoral approaches. The use of ADMs has played a key role in this shift, providing mechanical support and reducing tension on mastectomy flaps. Additional advancements, such as refined dissection strategies, perfusion assessment methods, and skin-sparing mastectomy techniques, have further contributed to the growing adoption of this method.

Nonetheless, several concerns continue to limit its broader implementation. These include the risks of inadequate flap perfusion, increased skin tension, and potential aesthetic drawbacks like rippling or implant visibility, particularly in patients with thin flaps or comorbid conditions.

In this context, we present the results of a 6-year retrospective study performed at a single institution, evaluating the outcomes of immediate prepectoral breast reconstruction without the use of ADMs. Our goal is to assess the safety and efficacy of this mesh-free approach in a large patient cohort and to examine how adjuvant radiotherapy may influence clinical outcomes. Patients were stratified based on their radiotherapy status to specifically analyze the impact of radiation exposure on complication rates and secondary procedures.

## Materials and methods

### Patients and methods

A retrospective analysis was conducted at a single institution, the Plastic Centre and Breast Unit of our Hospital, encompassing all patients who underwent mastectomy and prepectoral direct-to-implant breast reconstruction with silicone implants and without the use of ADM, between 2018 and 2023, included. Patient demographic, operative, and treatment variables were meticulously extracted for analysis.

This surgical option was extended to all patients meeting the following criteria: age between 25–70 years, BMI <30, surgical plans for nipple-sparing mastectomy (NSM) or skin sparing mastectomy (SSM), and absence of scheduled radiotherapy at the time of surgery.

The senior surgeons opted for the use of CPG Mentor™ highly cohesive anatomically shaped silicone-filled implants with a microtextured surface for the reconstructive procedures. This standardized approach reflects the institutional preference and contributes to consistency in outcomes across patient cases.

Unlike what is reported by the majority of studies found in the literature, we have extended this surgical option to patients with comorbidities or who are smokers, being confident in our well-established and detailed protocol of blunt mastectomy and in close collaboration between breast and plastic surgery teams.

For the aims of this study, we characterized patient characteristics including age, comorbidities, smoking status, type of mastectomy, and implant volume. This enabled us to observe postoperative complications across diverse clinical scenarios.

We documented postoperative occurrences of minor complications such as rippling, Baker capsular contracture grades 2 and seroma. Major complications included malrotation, Baker capsular contractures grades 3–4, hematomas, full-thickness skin necrosis, and implant loss from any cause.

During postoperative clinical follow-up visits, complications were identified and assessed by the senior surgeon. The severity of contracture was graded using the 4-grade Baker scale,^2^ while superficial skin necrosis was managed conservatively. Evaluation and management of all complications were overseen by the senior operating surgeon.

We stratified our patient population into two groups: the ones who did not undergo radiation therapy (group 1) and those who did (group 2). This decision was guided by the substantial impact of radiation therapy on breast tissue and reconstructive outcomes. Studies have consistently demonstrated that radiation therapy can lead to both immediate and delayed complications, including increased rates of capsular contracture and implant migration, as well as higher rates of complications overall.^3^, ^4^, ^5^, ^6^

### Statistical analysis

All statistical analyses were performed using the statistical system R version 4.0219 and the open-source statistical packages JASP v. 0.14.1 and Jamovi v. 2.0.20.

Categorical variables, such as, type of mastectomy, complications, secondary surgeries, BRCA mutation status, neoadjuvant and adjuvant therapies, radiotherapy status, smoking status, and comorbidities are presented in percentages while continuous variables such as age, follow-up duration, BMI, and implant size were summarized using means and standard deviations.

### Surgical technique of mastectomy

All mastectomies included in this study were performed by a dedicated team of three senior breast surgeons, while four experienced plastic surgeons were responsible for the reconstructive procedures.

A standard tumescent infiltration technique was employed during all mastectomies. The solution consisted of 20 mL of 1% lidocaine with epinephrine (1:100,000) diluted in 60 mL of sterile saline. It was administered into the subcutaneous plane using a 22-gauge spinal needle connected to a 20 mL syringe, with slow retrograde injection to ensure uniform distribution throughout the tissue.

Flap elevation was performed with meticulous sharp dissection using scissors, maintaining a plane superficial to the anterior mammary fascia to preserve the subcutaneous adipose layer. Cooper’s ligaments were carefully divided at their distal insertions. To minimize mechanical and thermal injury, self-retaining retractors were avoided, and frequent irrigation with cooled saline was used during dissection.

Subareolar tissue was excised in the subdermal plane to ensure complete glandular removal and to allow for intraoperative nipple biopsy. Once the flaps were dissected to the level of the pectoralis major fascia, electrocautery was utilized to separate the gland from the underlying muscle, allowing for complete resection of the parenchyma while preserving the integrity of adjacent structures.

Upon completion of the oncologic procedure, mastectomy flaps were inspected for viability. Particular attention was given to identifying signs of compromised perfusion, which would contraindicate immediate reconstruction. In cases where anatomical landmarks such as the lateral mammary fold or inframammary fold were disrupted during mastectomy, surgical correction was performed to restore proper definition of the breast pocket.

A temporary sizer was inserted into the prepectoral pocket to determine appropriate implant volume and to assess skin envelope adaptation. The patient was placed in an upright position on the operating table to verify flap perfusion and overall breast symmetry. Only in the presence of adequate vascularization was the decision made to proceed with definitive implant placement in a single-stage, prepectoral fashion.

It is worth noting that intraoperative evaluation of flap thickness remains subjective and poorly standardized. As such, consistent and reproducible methods for quantifying flap thickness would be beneficial for improving clinical decision-making.

Following flap assessment, the surgical field was re-prepared with povidone-iodine, sterile drapes were applied, and the surgical team changed gloves. The definitive implant was then positioned in the prepectoral space. Implants with high cohesivity were routinely selected to minimize the risk of upper pole rippling.

In cases of unilateral reconstruction, contralateral symmetrization procedures were discussed preoperatively and offered when appropriate.

Closed-suction drains were placed and subsequently removed once drainage volume fell below 50 mL over 24 h. Prophylactic antibiotics were administered prior to the induction of anesthesia, in accordance with institutional protocol.

## Results

Between 2018 and 2023, a total of 198 patients (239 breasts) underwent immediate prepectoral one-stage subcutaneous reconstructions without the use of acellular dermal matrix (ADM).

We stratified our patient population into those who underwent radiation therapy (RT, Group 2) and those who did not (Group 1).

In Group 1 there were 140 patients, 169 breasts with a mean follow up of 33 months, a mean age of 55.5 years, a mean BMI of 24.5; 28 active smokers (20%), 11 ex-smokers (7.9%), 29 had comorbidities (20.7%), 6 underwent previous therapy (4.3%) and 32 postmastectomy therapy (22.9%).

In contrast, in Group 2 there were 58 patients and 70 breasts with a mean follow up of 38.3 months, a mean age of 58.6 years, a mean BMI of 25.1; 10 active smokers (17.2%), 13 ex-smokers (22.4%), 8 had comorbidities (13.8%), 3 underwent previous therapy (5.2%), 11 postmastectomy therapy (19%). Results are summarized in Table 1.

**Table 1:** Clinical, intraoperative data.

Category	Group 1	Group 2
Number of patients, *n* (%)	140 (70.7%)	58 (29.3%)
Number of breasts, *n* (%)	169 (70.7%)	70 (29.3%)
Follow-up (months):	Mean = 33.0,	Mean = 38.3,
Mean, Standard Deviation (SD)	SD = 15.2	SD = 18.0
Age (years):	Mean = 55.5,	Mean = 58.6,
Mean, Standard Deviation (SD)	SD = 10.3	SD = 10.8
BMI (kg/m²): Mean, Standard Deviation (SD)	Mean = 24.5, SD = 4.7	Mean = 25.1, SD = 4.3
**Smokers, *n* (%)**		
Yes	28 (20.0%)	10 (17.2%)
No	101 (72.1%)	35 (60.3%)
Ex	11 (7.9%)	13 (22.4%)
Implant volume (cc):	Mean = 315.2,	Mean = 333.6,
Mean, Standard Deviation (SD)	SD = 99.7	SD = 99.5
**Mastectomy type, *n* (%)**		
Nipple sparing (NSM)	129 (92.1%)	54 (93.1%)
Skin sparing (SSM)	11 (7.9%)	4 (6.9%)
**Surgery Type, *n* (%)**		
Curative	127 (90.7%)	52 (89.7%)
Prophylactic	13 (9.3%)	6 (10.3%)
**Comorbidities, *n* (%)**		
Yes	29 (20.7%)	8 (13.8%)
No	111 (79.3%)	50 (86.2%)
**BRCA Mutations, *n* (%)**		
Yes	6 (4.3%)	3 (5.2%)
No	134 (95.7%)	55 (94.8%)
**Neoadjuvant Therapy, *n* (%)**		
Yes	6 (4.3%)	3 (5.2%)
No	134 (95.7%)	55 (94.8%)
**Adjuvant Therapy, *n* (%)**		
Yes	32 (22.9%)	11 (19.0%)
No	108 (77.1%)	47 (81.0%)

Among patients who did not undergo RT, comprising 140 individuals, complications included 4 cases of Baker grade 2 capsular contracture (2.3%), 2 cases of Baker grade 3 capsular contracture (1.2%), 10 cases of hematoma (5.8%), 12 cases of skin necrosis (7.1%), and 3 cases of seroma (1.8%).

Conversely, among the 59 patients who underwent radiation therapy, complications included 11 cases of Baker grade 2 capsular contracture (15.7%), 2 case of Baker grade 3 capsular contracture (2.9%), 1 case of Baker grade 4 capsular contracture (1.4%), 2 cases of hematoma (2.9%), 4 cases of skin necrosis (5.7%), 1 case of seroma (1.4%), and 2 instances of implant exposure (2.9%). Complications are summarized in Table 2.

**Table 2:** Complications.

	Group 1	Group 2
**Skin necrosis**	12 (7.1%)	4 (5.7%)
**Hematoma**	10 (5.8%)	2 (2.9%)
**Seroma**	3 (1.8%)	1 (1.4%)
**Capsular contracture**		
Grade 2	4 (2.3%)	11 (15.7%)
Grade 3	2 (1.2%)	2 (2.9%)
Grade 4	0 (0.0%)	1 (1.4%)
**Implant exposure**	0 (0.0%)	2 (2.9%)

Secondary surgical procedures were performed on 41 over 169 breasts in the non-radiation therapy group, including 11 lipofilling procedures (6.5%), 7 hematoma evacuation (4.1%), 4 implant removals (2.4%), 6 implant replacements (3.6%), and 12 surgical cleansings (7.1%).

In the radiation therapy group, secondary surgeries included 2 hematoma drainages (2.9%), 5 lipofilling procedures (7.1%), 2 DIEP flap reconstructions (2.9%), 4 implant removals (5.7%), 1 implant replacements (1.4%), and 2 surgical cleansings (2.9%). Table 3.

**Table 3:** Revisions (secondary surgeries).

	Group 1	Group 2
**Lipofilling**	11 (6.5%)	5 (7.1%)
**Implant replacements**	6 (3.6%)	1 (1.4%)
**Implant removal**	4 (2.4%)	4 (5.7%)
**Surgical cleansing**	12 (7.1%)	2 (2.9%)
**Hematoma evacuation**	7 (4.1%)	2 (2.9%)
**DIEP flap**	0 (0%)	2 (2.9%)

Fisher’s exact test indicated a statistically significant association between smoking and the occurrence of skin necrosis (*p* = 0.020). Radiotherapy was significantly associated with Baker grade 2 of capsular contracture (*p* < 0.0001). No other significant association was found between smoking or radiotherapy and any other complication. The results of the statistical analysis are shown in Table 4.

**Table 4:** Fisher’s exact test.

	Odds Ratio	*p*-Value
Skin necrosis	0.49	0.22
Hematoma	0.42	0.17
Seroma	0.39	0.61
Capsular contracture		
Grade 2	0.14	0.001
Grade 3	0.60	1.00
Grade 4	0.00	1.00
Malrotation	0.00	1.00
Infection	0.00	1.00

## Discussion

The introduction of silicone breast implants in the early 1960s marked the beginning of implant-based breast reconstruction, initially performed in a delayed setting. It was only later that immediate reconstruction following mastectomy began to gain acceptance. Despite this progress, early attempts at prepectoral implant placement were largely abandoned in the 1980s due to unacceptably high complication rates, particularly with respect to capsular contracture, skin flap necrosis, and poor aesthetic outcomes.^7^ As a result, the submuscular approach became the preferred technique for many years.

However, submuscular implant positioning has shown significant limitations over time, including postoperative pain, animation deformities, and difficulties in accommodating fixed-volume implants due to the constraints of the pectoralis muscle. These issues led to widespread adoption of two-stage reconstruction using tissue expanders as a safer alternative.

In response to the drawbacks of total submuscular placement, the dual-plane technique was developed, offering partial implant coverage by the pectoralis major muscle superiorly and the skin flap inferiorly. Although this approach improved lower pole expansion, it often resulted in excessive descent of the implant, also known as “bottoming out”.^8^^,^^9^

By the mid-2000s, the use of acellular dermal matrices (ADMs) or synthetic mesh helped refine the dual-plane method. ADMs provided additional support to the lower pole and lateral implant surface, allowing for improved implant stability and pocket control. Despite these advantages, concerns remained regarding functional outcomes, such as shoulder mobility and animation deformity due to muscle detachment.^10^^,^^11^

To address these limitations, the prepectoral approach was reintroduced—this time aided by ADM or synthetic mesh-enabling complete subcutaneous implant placement without disturbing the pectoralis major or serratus anterior muscles. This technique offers reduced morbidity, improved cosmesis, and preservation of shoulder function. It is now commonly performed as a one- or two-stage procedure depending on the condition of the mastectomy flaps and desired breast volume.^12^

The clinical advantages of prepectoral implant placement are well recognized. Muscle preservation leads to decreased postoperative pain, elimination of animation deformity, shorter recovery times, and often a more natural breast contour. Moreover, placing the implant in the anatomical location of the native breast improves overall aesthetic satisfaction.^13^ The technique has become increasingly popular in the context of minimally invasive, patient-centered reconstructive strategies.^14^

While the use of ADM has supported the broader adoption of the prepectoral approach, its necessity remains a topic of ongoing debate. Advances in implant technology and surgical technique have led some authors to question whether ADM is always required. Delong et al., for instance, reported improved outcomes and reduced capsular contracture rates associated with silicone implants alone, even before ADM became widespread.^15^

Similarly, Nolan and colleagues conducted a meta-analysis that found no significant increase in complication rates in prepectoral reconstructions performed without ADM when compared to those using ADM, reinforcing the viability of mesh-free techniques.^16^ However, substantial clinical data on direct-to-implant prepectoral reconstruction without ADM remain limited. Among the few published series, Urban et al. presented favorable results but emphasized the need for further studies involving larger cohorts.^17^

In light of this, we undertook a 6-year retrospective analysis of prepectoral direct-to-implant breast reconstructions performed without ADM. Our findings demonstrate that, in selected patients, this approach can be both safe and effective. Notably, complication rates were low among patients who did not receive radiotherapy, with capsular contracture and other adverse events occurring at acceptable frequencies. These results suggest that omitting ADM may not compromise outcomes in appropriately selected non-irradiated patients.

However, our data also highlight the impact of radiotherapy on surgical outcomes. Patients who underwent adjuvant radiation showed significantly higher rates of grade 2 capsular contracture, in line with findings reported in the literature. This underscores the need for careful patient selection and tailored surgical planning in the setting of postmastectomy radiotherapy.

When comparing our results to those of studies involving ADM, it becomes evident that while ADM may offer certain benefits-such as reducing the risk of capsular contracture—it also introduces additional costs and potential complications, including infection and seroma formation. As such, the decision to use ADM should be individualized, taking into account patient anatomy, comorbidities, and surgeon experience.

In a recent meta-analysis, Awadeen et al. reported a fivefold increase in the risk of capsular contracture among irradiated patients undergoing prepectoral reconstruction with ADM (RR 5.17).^18^ Although our study did not include ADM, the observed risk of grade 2 contracture was similarly elevated in the irradiated subgroup. Importantly, no increase was observed in higher-grade contracture, suggesting that even in the absence of ADM, outcomes may remain acceptable under certain conditions.

Our results add to the growing body of literature supporting the use of prepectoral breast reconstruction without ADM. By demonstrating comparable results in terms of complication rates and need for secondary procedures, our study supports the idea that this mesh-free approach can be a cost-effective and clinically sound alternative in select populations. This may be particularly relevant in healthcare settings where cost containment is a priority.

In conclusion, our study offers meaningful evidence that immediate prepectoral reconstruction without ADM can be performed safely and effectively, particularly in non-irradiated patients. While additional research is necessary to validate these findings and to directly compare mesh-assisted and mesh-free outcomes, our results suggest that the latter should be considered a valuable and versatile option within the reconstructive surgeon’s armamentarium.

## Funding

None.

## Declaration of competing interest

None declared.
